# Prenatal and early life exposure to air pollution induced hippocampal vascular leakage and impaired neurogenesis in association with behavioral deficits

**DOI:** 10.1038/s41398-018-0317-1

**Published:** 2018-11-29

**Authors:** N. C. Woodward, A. Haghani, R. G. Johnson, T. M. Hsu, A. Saffari, C. Sioutas, S. E. Kanoski, C. E. Finch, T. E. Morgan

**Affiliations:** 1 Leonard Davis School of Gerontology, University of Southern California, Los Angeles, CA USA; 2 Human and Evolutionary Biology Section, Department of Biological Sciences, University of Southern California, Los Angeles, CA USA; 3 Neuroscience Program, University of Southern California, Los Angeles, CA USA; 4 Viterbi School of Engineering, University of Southern California, Los Angeles, CA USA; 5 Dornsife College, University of Southern California, Los Angeles, CA USA

## Abstract

Exposure to traffic-related air pollution (TRAP) is associated with a range of neurodevelopmental disorders in human populations. In rodent models, prenatal TRAP exposure increased depressive behaviors and increased brain microglial activity. To identify cellular mechanisms, we examined adult neurogenesis and the blood–brain barrier (BBB) in relation to cognition and motivated behaviors in rats that were exposed to a nano-sized TRAP subfraction from gestation into adulthood. At age 5 months, exposed male rats had 70% fewer newly generated neurons in the dentate gyrus (DG) of the hippocampus. Microglia were activated in DG and CA1 subfields (35% more Iba1). The BBB was altered, with a 75% decrease of the tight junction protein ZO-1 in the CA1 layer, and twofold more iron deposits, a marker of microhemorrhages. The exposed rats had impaired contextual memory (novel object in context), reduced food-seeking behavior, and increased depressive behaviors (forced swim). Deficits of de novo neurogenesis were inversely correlated with depressive behavior, whereas increased microbleeds were inversely correlated with deficits in contextual memory. These findings give the first evidence that prenatal and early life exposure to TRAP impairs adult hippocampal neurogenesis and increases microbleeds in association with behavioral deficits.

## Introduction

Traffic-related air pollution (TRAP) is a ubiquitous environmental health hazard, with increasingly recognized neurodevelopmental effects. Gestational TRAP exposure alters cognitive development, delays myelination, and increases anxiety and depressive behaviors^1–4^. Autism spectrum disorders are also associated with increased by elevated TRAP exposure during pregnancy and early postnatal life^5–7^. Rodent models of gestational or early postnatal TRAP exposure show impaired object recognition, increased impulsivity, and depressive behavior^8–10^.

Adult brain neurogenesis has not been assessed for the developmental impact of TRAP. Because depressive behavior is correlated with de novo neurogenesis^11^, we hypothesized that prenatal and early life TRAP exposure would impair hippocampal neurogenesis and hippocampal-dependent behavior, in association with increased brain inflammatory responses. Blood–brain barrier (BBB) leakage and microglial activation were indicated for a small sample of young adults exposed to severe air pollution in Mexico City^12^. Microglia were also activated in white matter and hippocampal regions in 2-month-old offspring from TRAP-exposed mothers^8^.

The present study extended gestation exposure^9^ into the young adult phase, to better model the human experience. Our exposure model uses the smallest sizes of particulate matter, which are of high relevance to the brain because their small size allows penetrance^13^. We examined hippocampal de novo adult neurogenesis together with microglial activation, BBB tight junction marker zona occludens protein 1 (ZO-1), iron deposition, and hippocampal-dependent behaviors. We focused on hippocampal subfields because of CA1 neuronal vulnerability in adult mouse TRAP exposures^14,15^, which models the CA1 vulnerability in Alzheimer disease^16^.

## Materials and methods

### Experimental design

**Fig. 1:**
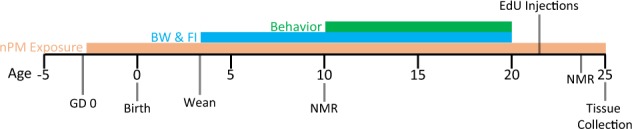
Experimental schedule. Rats were exposed to nPM throughout gestation and continued for male offspring from birth until 25 weeks of age. Body weight (BW) and food intake (FI) were measured from weaning until 20 weeks. Body mass composition was measured at 10 and 24 weeks. Behaviors were tested from 10 to 20 weeks. EdU was injected i.p. 2 weeks after behavioral testing concluded; brains were collected 3 weeks later

The neurocognitive effects of prenatal and early life exposure to air pollution were tested by exposing male rats to concentrated ambient air pollution for 28 weeks. An experimental timeline is given in Fig. 1. Rats were exposed 5 h/day, 3 days/week beginning gestational day 2, through gestation, until 25 weeks of age. For gestational exposure, six pregnant dams per experimental group were randomly placed into control or nPM groups, yielding 36 and 38 pups, respectively. At weaning, female rats and moms were removed from the exposure regiment due to space constraints of the exposure chambers. Sample size was the maximum number of male rats able to fit into the exposure chamber, with excluded females being a limitation of the current study. Weekly body weight and food intake measurements began at weaning and continued for 16 weeks. Body composition was analyzed by nuclear magnetic resonance (NMR) immediately prior to behavioral assessment at 10 weeks, and at 25 weeks immediately prior to tissue collection. Behavioral testing began at 10 weeks of age, and continued until 20 weeks of age. Three weeks prior to tissue collection, rats were injected with the thymidine analog 5-ethynyl-2′-deoxyuridine (EdU) to measure neurogenesis. At 25 weeks of age, brains were processed for histochemistry.

### Animals and ethics statement

Sprague-Dawley pregnant rats (gestational day 0) were obtained from Envigo (Hayward, CA; RRID:RGD 1566457). Protocols were approved by the University of Southern California Institutional Animal Care and Use Committee. Rats were maintained under standard conditions with free access to rodent chow and water according to NIH and IACUC guidelines. TRAP exposure did not alter body weight and food intake in weekly assessments. Rats were euthanized by ketamine (90 mg/kg), xylazine (2.8 mg/kg), and acepromazine (0.72 mg/kg).

### Particulate matter collection and exposure

The nanoscale particulate matter (nPM) in these studies are a subfraction of ultrafine TRAP-PM_0.2_ ( < 200 nm diameter) collected near the CA-110 Freeway in Los Angeles^9,17^. These aerosols represent a mix of ambient particulate material (PM) mostly from vehicular traffic^18^ and comprise 20% by mass of ambient PM_2.5_ in that location^19^. PM_0.2_ were collected on Teflon filters, resuspended in deionized water by sonication, and re-aerosolized for exposures^20^. The resuspension is designated as nPM in distinction from ultrafine PM, because it represents the water-soluble subfraction of total PM0.2, which includes water-soluble metals and organic compounds that are efficiently transferred from filters into aqueous suspension. Relative to the total filtered-trapped ultrafine PM, the nPM subfraction is depleted in black carbon (BC) and water-insoluble organic compounds, including most polycyclic aromatic hydrocarbons (PAHs)^20,21^. The nPM include various water-soluble organic carbons (WSOCs), which correlate with overall redox activity of nPM^22^. Endotoxin levels are below threshold by *Limulus* amebocyte assay; frozen stocks at −20 °C retain chemical stability for > 30 days^20,21^. By in vitro assay for tumor necrosis factor alpha (TNF-α) release, current nPM batches have equivalent activity to those of prior years. Rats were exposed for 5 h/day at 340 µg/m^20^, 3 day/week from gestation day 2 until 25 weeks of age. Control mice were exposed to high efficiency particulate air (HEPA) filtered particulate-free air under the same conditions.

### Behavior

Behavioral testing begun at 10 weeks of age. Rats were transitioned to reverse light cycle housing prior to differential reinforcement of low response rates (DRL) behavior testing, and maintained for the duration of the experiment. All behavior equipment was thoroughly cleaned with 10% aqueous ethanol water after every rat’s trial, before beginning the next rat’s trial.

#### Differential reinforcement of low rates of response (DRL)

DRL was performed to examine food-seeking behavior (all DRL sessions) and impulsivity (DRL20 specifically). Rats were kept on reverse light cycle housing, and tested at the onset of darkness. Operant conditioning for DRL was performed in Med Associates conditioning boxes (Fairfax, VT). Rats were placed on a DRL paradigm, where DRL5, -10, and -20 represent the delay in seconds between reinforcements. Each session lever presses are rewarded only if the required time had elapsed since the prior press; lever presses before the timeout period had elapsed reset the timeout. DRL was conducted over 24 1-h sessions, each on a separate day. Rats began on DRL0 (zero second timeout) for five sessions to train for palatable food (45 mg pellet, 35% kcal enriched with sucrose, #F05989, Bio-serv) paired with 1-s light stimulus. Rats pressed on average 110 times during the 1-h period by the end of DRL0. Reinforcement delays (time outs) progressed to DRL5 (sessions 1–5), then DRL10 (sessions 6–10). Finally, DRL20 for sessions 11–20 to test impulsivity behavior^23,24^, which was recorded as efficiency in obtaining food rewards (rewards obtained divided by lever presses). Four extinction trials followed DRL where no reward was delivered following lever presses.

#### Novel object in context (NOIC) recognition

Hippocampal-dependent object and context recognition was assessed by NOIC^25^. Rats were habituated to novel object recognition chambers (31.8 × 48.3 × 51.8 cm) in two separate locations for 5 min on days 1 and 2. On day 3, rats were exposed to two distinct objects (A and B) for 5 min in the first location. Objects were cylindrical jars filled with blue water, and square transparent glass containers (7 × 8.3 × 10.2 cm). Exploration was defined as touching objects with whiskers, nose, or forelegs. Close proximity and standing on the object was not counted as exploration. Rats showed no preference between objects on day 3, and objects A and B were counterbalanced between trials. On day 4, rats were placed in location two, with duplicates of object A, again for 5 min. On day 5, rats were placed again in location two, this time with objects A and B. Time spent investigating the object was recorded, with blinded identification on live video feed. The discrimination index is the exploration time of object B on the final day, divided by total time spent exploring objects.

#### Elevated zero maze

Anxiety behavior was tested using an elevated zero maze (63.5 cm fall height, 117 cm outside diameter), with a circular track, divided into four sections. Two sections were open, with 3 cm high curbs, and two sections were closed, with 17.5 cm high walls. Rats were observed in the maze for 5 min for total time spent in open sections (defined as the head and front two paws in open arms), and total open arm entries.

#### Forced swim

Rats were placed in a cylindrical water bath (41 cm height, 26 cm wide). Water depth was 18 cm at 24–25 °C. Trials were video recorded for 5 min and blindly scored for latency to first period of immobility and total time immobile recorded.

### Inflammatory multiplex assay

Serum protein concentration of CXCl1, IFN-γ, IL-1 β, IL-4, IL-5, IL-6, IL-10, IL-13, and TNF-α inflammatory proteins were analyzed by the V-PLEX Proinflammatory Panel 2 immunoassay (K15059D-1, Mesoscale Diagnostics, Rockville, MD). Serum was collected by cardiac puncture prior to perfusion, and blood was centrifuged at 2000 *g* for 20 min at 4 °C. Serum was removed and stored at −80 °C until use. The plate was blocked with proprietary blocking solution for 1 h then washed three times. Samples were added and allowed to incubate at room temperature for 2 h. Plate was then washed, detection antibody added, and incubated for 2 h. Plate was washed three times, read buffer was added, and plate was read on the MESO QuickPlex SQ 120 (Mesoscale Diagnostics, Rockville, MD). Statistical analysis was performed using *t*-test.

### Histochemistry

Whole-body perfusion with 0.9% saline was followed by 4% paraformaldehyde in 0.1 M borate buffer (pH 8.5). Brains were cryoprotected in 12% sucrose and frozen in isopentane for coronal sectioning (30 µm). Six sections (Bregma −3.00 to −3.80) were analyzed per brain for all endpoints.

#### EdU

Adult rats were injected intraperitoneally (i.p.) with 41 mg/kg EdU (molar equivalent to 50 mg/kg BrdU, bromodeoxyuridine) seven times over 3 days, 18 days before tissue collection. EdU, a thymidine analog, was chosen over the traditional thymidine analog BrdU, because EdU detection yields higher quality sections by not requiring heat or acid treatment^26^. Sections were co-immunostained for EdU (Alexa Fluor 555 Imaging Kit, Thermo Fisher Scientific) and cell markers (glial fibrillary acidic protein (GFAP), ionized calcium binding adaptor molecule 1 (Iba1), and neuron nuclear antigen (NeuN)).

#### Immunofluorescence

Primary antibodies were from Abcam: NeuN (1:10,000 concentration, ab104224, RRID:AB_10711040); GFAP (1:1000, ab7260, RRID:AB_305808); Iba1 (1:1000, ab5076, RRID:AB_91676); ZO-1 (1:5000, ab59720, RRID:AB_946249). ZO-1 was colocalized to lectin (Vector Labs, DL-1174, RRID:AB_1336404).

#### Iron deposits

Microbleeds were detected as hemosiderin (iron) deposits by Perls’ Prussian blue histochemistry^27^. Cerebral microbleeds were scored for number and size. Quantification was by blinded analysis; microbleed area was manually outlined.

### Statistical analysis

Data were analyzed by repeated-measures analysis of variance (ANOVA) for operant conditioning; linear regression for correlation analysis; and two-tailed *t-*test for other behavioral tests and histochemistry. Statistical variance is depicted in all graphs. Pairwise Pearson correlation and associated significance adjusted by Holm’s method for multiple testing were calculated by “Psych” package in R studio. Rats with missing measurements were excluded from correlational analysis. Missing measurements in behavioral tests can occur from not learning the reward paradigm in DRL, and not exploring novel object in the first test day of NOIC. Missing measurements for histochemical analysis occurred only if there were suboptimal sections from sliced brains restricting regional analysis. *N* = 15–17 for behavioral tests, and 13–16 for histochemical analysis. Treatment groups were blinded during analysis of behavioral testing and histochemistry.

## Results

Male rats were exposed to filtered air or to nPM, a nanoscale subfraction of TRAP, during gestation up through young adulthood (25 weeks) (Fig. 1). Behavioral studies began at 10 weeks; brains were analyzed at 25 weeks of age.

### Body mass, food intake, and circulating cytokines

Exposure to nPM did not obviously alter postnatal health or somatic development, assessed by body weight and food intake (Supplementary Figure 1A, B). Total fat mass was 10% lower at 10 and 24 postnatal weeks (Supplementary Figure 1C), without change in lean body mass or fluid mass (t(32) = 2.544, *p* = 0.0160; t(30) = 2.120, *p* = 0.0424, respectively) (Supplementary Figure 1D, E). Serum cytokines responded selectively to nPM exposure: three anti-inflammatory cytokines decreased by 20% (IL-4, t(30) = 3.558, *p* = 0.0013; IL-10, t(30) = 3.284, *p* = 0.0026; IL-13, t(30) = 2.459, *p* = 0.0199) (Supplementary Figure 2A, B, C), whereas pro-inflammatory cytokines were unchanged (IFN-y, IL-1b, IL-5, IL-6, CXCL1, TNF-α; data not shown).

### Neurogenesis and astrogenesis

**Fig. 2:**
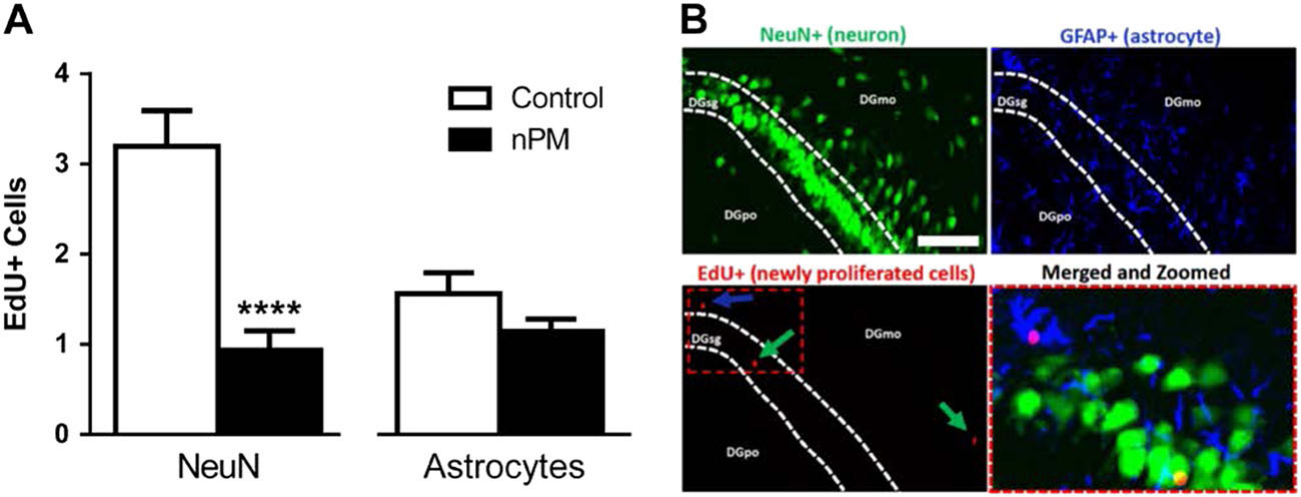
Neurogenesis, but not astrogenesis, was decreased by nPM exposure in the DG. **a** Neurogenesis was decreased 70% (*p* < 0.0001). Astrogenesis was unchanged. **b** Representative images of EdU staining in the granule cell layer of the DG. Clockwise, from upper left: NeuN staining; GFAP staining; EdU-positive cells, arrows show neurons in green (NeuN) and astrocytes in blue (GFAP); colocalization of EdU-positive cells with GFAP and NeuN immunostaining. Scale bar = 100 μm. Mean ± SEM; ****p < 0.0001. DG dentate gyrus, EdU 5-ethynyl-2′-deoxyuridine, GFAP glial fibrillary acidic protein, MO molecular layer, NeuN neuron nuclear antigen, PO polymorphic layer, GCL granule cell layer

Exposure to nPM impaired neurogenesis assessed by EdU in select brain regions in young adults. In the hippocampus, neurogenesis was decreased by 70% in the dentate gyrus (DG), whereas astrogenesis was not altered (t(25) = 4.896, *p* < 0.0001; Fig. 2a). The colocalization of EdU with cell-specific markers is shown in Fig. 2b: NeuN, neurons; GFAP, astrocytes. In the subventricular zone (SVZ), EdU was not colocalized with NeuN or GFAP, nor were the numbers of EdU-positive cells altered by nPM exposure (data not shown).

### Microglial activation

**Fig. 3:**
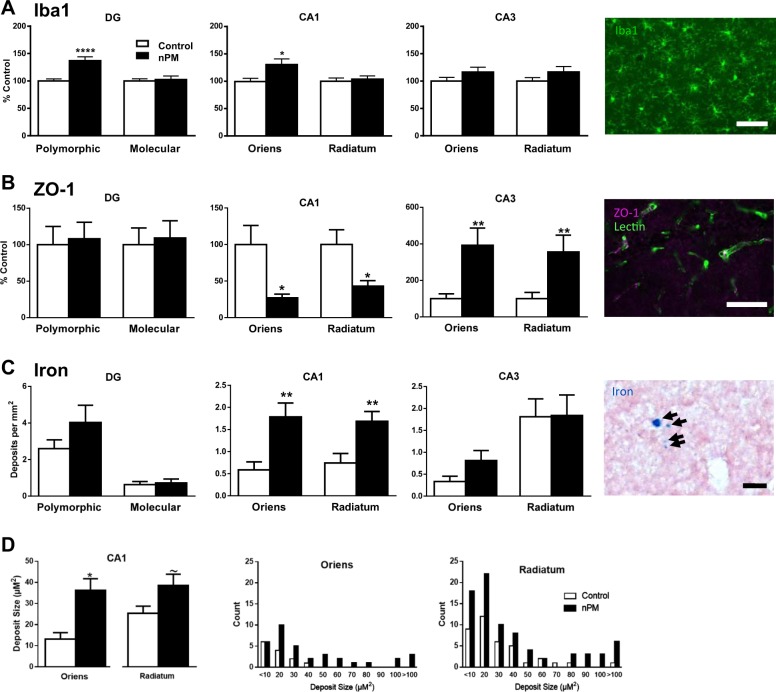
Exposure to nPM activated microglia and altered endothelial tight junctions, with differences in hippocampal subregions. subregions **a** Microglial Iba1 immunostained area: DG polymorphic layer ( + 35%, *p* < 0.0001); no change in DG molecular layer. CA1 stratum oriens ( + 30%, *p* < 0.05); s. radiatum, no change. CA3, no change. Far right, representative image of Iba1 staining in CA1 stratum radiatum. Scale bar, 50 μm. **b** Tight junctions of vascular endothelial cells were assessed by immunostaining of ZO-1 (zona occludens protein). ZO-1 values are normalized to lectin levels, which was not altered by nPM exposure (data not shown): DG: no nPM effect; CA1 s. oriens (−70%, *p* < 0.05) and s. radiatum (−40%, *p* < 0.05); CA3: twofold increase in s. oriens (*p* = 0.003) and s. radiatum (*p* < 0.01). Far right, representative ZO-1 staining (purple) counterstained by lectin (green) in CA1 s. radiatum. Scale bar, 100 μm. Mean ± SEM; *p < 0.05, **p < 0.01; ****p < 0.0001. **c** Iron deposits (microbleeds) by Perls’ Prussian blue staining of hemosiderin in hippocampus subregions: DG, no nPM effect; CA1 layers: nPM, + 300% in s. oriens (*p* < 0.01) and + 240% in s. radiatum (*p* < 0.01); CA3, no nPM effect; far right, microbleeds in s. radiatum. Scale bar, 20 μm. **d** Iron deposit size: hemosiderin stained area increased 270% by nPM in s. oriens (*p* < 0.05), with a trend toward increase in s. radiatum (*p* < 0.10). Frequency distributions of deposit size in s. oriens and s. radiatum. Mean ± SEM; control, *p < 0.05; ~*p* < 0.10

Microglial activation (Iba1 total immunostained area) was increased by nPM exposure in specific hippocampal subregions. In the DG polymorphic layer, Iba1 increased 35% (t(28) = 4.841, *p* < 0.0001), but did not change in the DG molecular layer (Fig. 3a, panel 1). In the CA1 neuropil layer, nPM increased Iba1 by 30% in stratum oriens (t(28) = 2.682, *p* = 0.0121; Fig. 3a, panel 2), but did not change in CA1 stratum radiatum and CA3 (Fig. 3a, panel 3). Far right, representative Iba1 immunostaining is depicted.

### BBB tight junctions

Because of indications that air pollution can alter tight junctions in cerebrovascular endothelial cells^28^, we assessed the zona occludens protein (ZO-1), a marker for tight junctions. The immunostained area of ZO-1 showed hippocampal subregional selectivity of nPM exposure (Fig. 3b). In the DG, ZO-1 was unaffected by nPM, whereas CA1 s. oriens and radiatum ZO-1 decreased by 70% (t(25) = 2.468, *p* = 0.0208 and t(28) = 2.503, *p* = 0.0184, respectively) (Fig. 3b). In contrast, in the CA3 layer, the s. oriens and s. radiatum had > 2-fold increases (t(27) = 3.280, *p* = 0.0029 and t(27) = 2.797, *p* = 0.0094, respectively) (Fig. 3b). Far right, representative ZO-1 immunostaining.

### Iron deposits

To evaluate the impact of ZO-1 changes on the BBB, cerebral microbleeds were assessed by hemosiderin staining. Hippocampal subregions responded differentially to nPM. CA1 had twofold more iron deposits in s. oriens and s. radiatum (t(19) = 3.771, *p* = 0.0013 and t(19) = 3.274, *p* = 0.0040, respectively), whereas DG or CA3 did not show definitive change (Fig. 3c). Frequency distributions showed increased area of iron deposits by nPM exposure in CA1 s. oriens s. oriens (t(46) = 2.522, *p* = 0.0152), and a trend in s. radiatum (t(115) = 1.1634, *p* = 0.1050), with greater upper range without change in modal size (Fig. 3d).

### Food-seeking behaviors by differential reinforcement of low response rates (DRL)

**Fig. 4:**
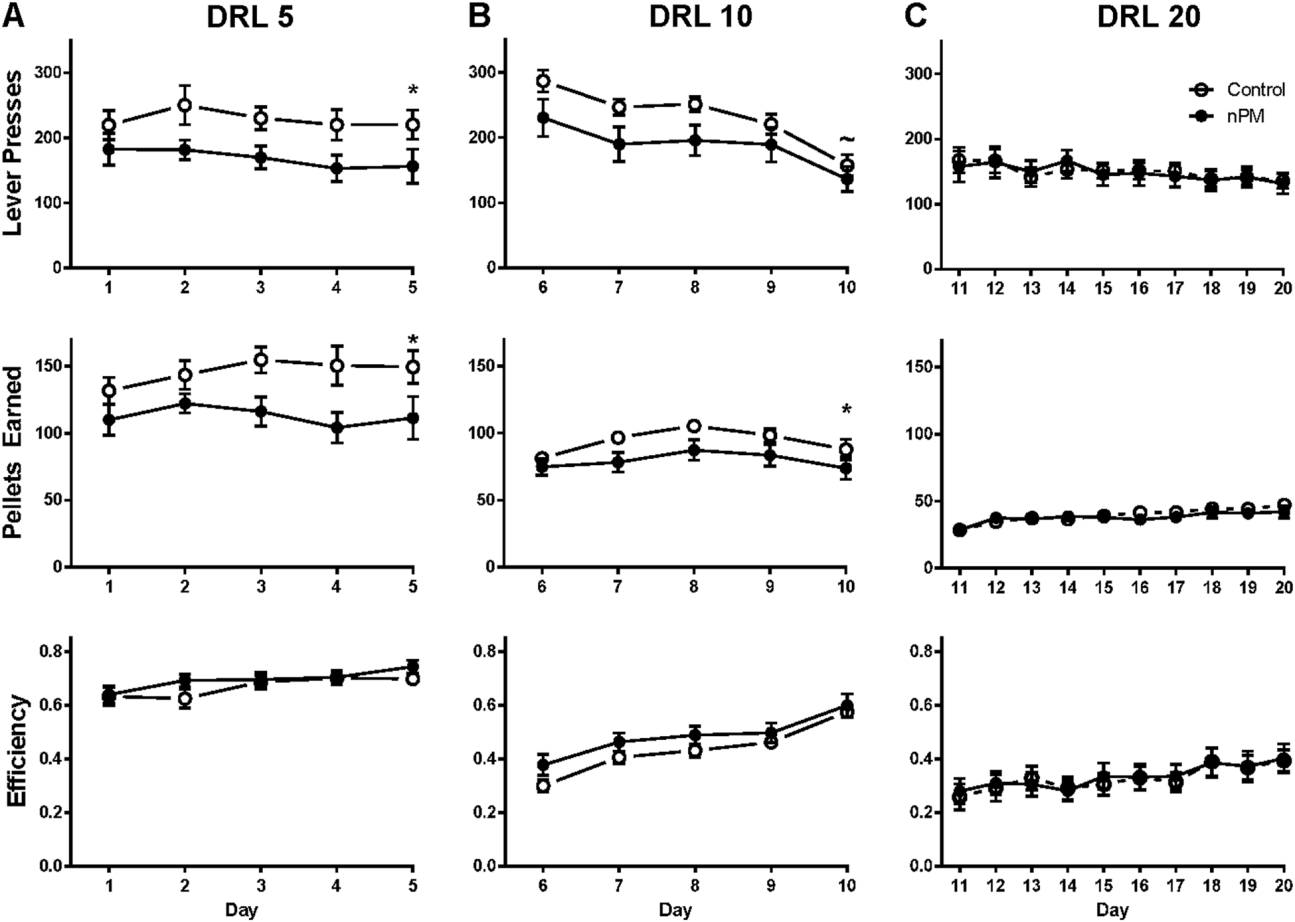
Differential reinforcement of lower rates of behavior (DRL) test, with timeout intervals increasing from 5, 10, and 20 s. **a** nPM exposed mice showed decreased food-seeking behavior, with decreased lever presses (*p* < 0.05, top panel). Decreased lever presses corresponded with decreased pellets earned per session (*p* < 0.05, middle panel). Efficiency did not change by nPM (bottom panel), defined by the number of pellets earned divided by number of lever presses. **b** Decreased food-seeking behavior was marginally altered by nPM in the DRL10 schedule, with fewer lever presses (~ *p* = 0.055, top panel), and fewer pellets earned (*p* < 0.05, middle panel) for nPM exposure. Efficiency was not altered (bottom panel). **c** DRL20 did not show nPM effects. Mean ± SEM; repeated-measure ANOVA, *p < 0.05, ~*p* = 0.055

Rats exposed to nPM had impaired food-seeking behavior (Fig. 4). The designations DRL5, 10, and 20 refer to sequentially increased experimental delays in seconds between reinforcements (Materials and methods). For DLR5 and DLR10, the nPM exposed rats made consistently fewer lever presses and fewer pellets earned. In DRL5, nPM exposure accounted for 11.49% and 12.97% of the total variation for lever presses and pellets earned (repeated-measures ANOVA, *F*(1, 26) = 5.215, *p* = 0.0308 and *F*(1, 26) = 5.529, *p* = 0.0266). In DRL10, nPM exposure accounted for 7.768% and 9.871% of the total variation for lever presses and pellets earned (*F*(1, 26) = 4.425, *p* = 0.0452, *F*(1, 26) = 4.027, *p* = 0.0553, respectively). No changes were seen for active lever presses in DRL0. Other related behaviors were unaltered: food-seeking behavior in DRL20 (Fig. 4c, top and middle panels); and impulsivity (Fig. 4c, bottom panel), measured by efficiency in the DRL20 paradigm. Nor did nPM exposure alter learning, measured by efficiency during acquisition (Fig. 4, bottom panels) or by extinction (data not shown).

### Hippocampal-dependent memory (NOIC)

**Fig. 5:**
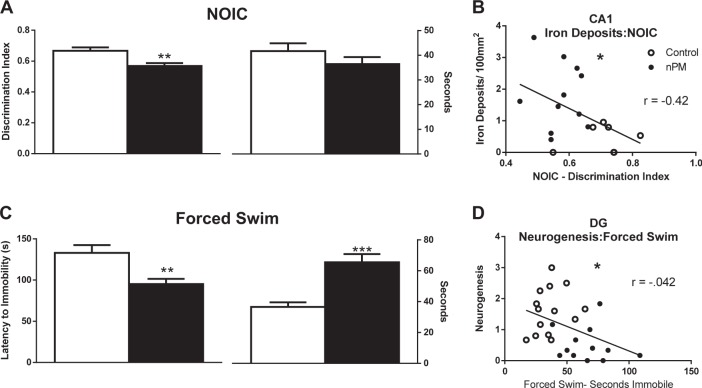
nPM exposure impaired hippocampal-dependent contextual memory and increased depressive behaviors. hippocampal-dependent **a** Left panel: impaired contextual memory was observed by decreased discrimination index for a novel object in context (NOIC) by 15% (*p* < 0.01). Right panel: NOIC, no change in total object exploration. **b** Inverse correlation between iron deposits in the CA1, and performance on NOIC (*r* = −0.48, *p* < 0.05). **c** Left panel: depressive behavior measured by the forced swim test. nPM exposure decreased latency to first period of immobility by 30% (*p* < 0.01). Right panel: in the forced swim test, nPM exposure increased total time immobile by 80% (*p* < 0.01). **d** Inverse correlation between neurogenesis in the DG and total time immobile in the forced swim test (*r* = −0.42, *p* < 0.05). Mean ± SEM; control, *p < 0.05; **p < 0.01; ***p < 0.001

Exposure to nPM impaired hippocampal-dependent memory, assessed by impaired contextual memory for a known object in a novel context (NOIC). Exposed rats had 15% decreased discrimination (t(30) = 3.405, *p* = 0.0019; Fig. 5a, left panel), with no effect on overall object exploration (right panel). These deficits in contextual memory showed inverse correlation with increased microbleeds in CA1 (*r* = −0.48, linear regression *p* < 0.0446, Fig. 5b).

### Depressive behaviors (forced swim)

Depressive behavior in the forced swim test, quantified by the onset of immobile behavior and time immobile and also viewed as a measured of adaptive vs. passive behavior, were increased by nPM exposure (Fig. 5c): decreased latency to onset of immobility by 30% (t(28) = 3.126, *p* = 0.0041; left panel), and increased total immobility by 80% (t(28) = 5.054, *p* < 0.001; right panel). The duration of immobility in the forced swim test varied inversely with neurogenesis in the DG (*r* = −0.42, linear regression *p* = 0.0362, Fig. 5d) and directly with microglial activation in the stratum oriens of the CA1 (*r* = 0.505, linear regression *p* = 0.0083).

### Anxiety behavior (elevated zero maze)

Anxiety behavior was not altered by nPM exposure in the elevated zero maze (Supplementary Figure 3). Time spent in open area (left panel), and total number of entries into the open areas (right panel) were equal between nPM exposed and control groups.

## Discussion

Prenatal and early life exposure of male rats to TRAP (TRAP-nPM) from gestation into maturity showed novel changes of the BBB with increased microbleeds and altered tight junction proteins in subfields of the hippocampus, and impaired hippocampal neurogenesis. Behavioral changes included impaired hippocampal-dependent contextual memory and increased depressive responses. The impairments of neurogenesis showed inverse correlation with depressive responses. Although deficits in contextual memory showed inverse correlation with increased microbleeds. Nonetheless, the prolonged exposure to high traffic levels of ultrafine PM did not alter litter size, or postnatal growth of lean body mass. These novel findings give insight into human neurodevelopmental deficits from urban air pollution associated with increased risk of autism spectrum disorders, affective disorders, and impair cognitive development^1,2,4,6,29,30^.

The present rodent model expanded studies of neurodevelopmental exposure to air pollution particles that did not extend from gestation into early adulthood pollution from this^9^ and other laboratories^31–34^. By including pre- and postnatal developmental phases, the present model may more closely represent human developmental exposures to TRAP that are associated with behavioral and cognitive dysfunctions.

BBB alterations from air pollution were indicated in small samples of young adults from a highly polluted Mexican city^12^. The present exposure model shows definitive increase of microbleeds and deficits of the tight junction protein ZO-1 in specific hippocampal subregions: ZO-1 was decreased in the CA1, but increased in the CA3 layer, and was unchanged in the DG. The 70% decrease of ZO-1 in CA1 is thus consistent with the twofold increase of microbleeds in CA1. Correspondingly, in the CA3, levels of ZO-1 increased, but without change in microbleeds. These associations with microbleeds predict that the TRAP-induced increase of BBB permeability in ApoE-knockout mice^35^ would also be accompanied by increased microbleeds. The increase of microbleeds extends histochemical findings of “iron deposition” in the corpus callosum of gestationally exposed mice; other brain regions were not reported^31^. The early onset of cerebral microbleeds from prenatal and early life TRAP exposure in this rodent model reveals that little is known of microbleeds in human developmental exposure to TRAP. Because microbleeds are associated with accelerated cognitive decline^36^ and increased risk of dementia^37^, these rodent findings suggest that early human exposure to TRAP may add a premature burden of microbleeds that increase dementia risks.

Prenatal and early life exposure to TRAP also impaired performance on the forced swim test, corroborating findings of impaired tail suspension performance after gestational exposure of mice^9^. The forced swim and tail suspension tests indicate depressive behaviors, and are used to evaluate anti-depressant drugs^38^. In the present study, TRAP exposure did not alter impulsivity (DRL20) or anxiety behaviors (zero elevated maze), which depend less on neurogenesis. Prenatal only exposure to diesel exhaust did not alter anxiety, unless paired with a postnatal high fat diet^10^, whereas postnatal exposure to TRAP increased impulsivity^39^.

The 70% reduction of neurogenesis in the adult hippocampal DG from nPM exposure was inversely correlated with the time immobile in forced swim test. This depressive response association is consistent with impaired hippocampal neurogenesis from stress in young adult rodents, also associated with depressive behavior^40,41^. Even unstressed young mice showed correlations of neurogenesis with exploratory activity^42^. Although postnatal neurogenesis may decline faster in humans during postnatal stages than in rodents^43^, rodent models remain valuable for testing of interventions to protect brain development from air pollution.

The observed reduction of neurogenesis in TRAP-exposed young adult rats may share developmental defects from gestational TRAP exposure. Our prior study showed impaired cortical neuron differentiation in vitro from primary cultures of cerebral cortex neurons obtained from gestation-only TRAP-nPM exposure^9^. Future studies could examine the impact of gestational TRAP exposure on radial glia that generate neurons for cerebral cortex and on the later developing DG of the hippocampus. In young adult brains, neurogenesis is prominent in two regions: the subgranular zone (SGZ) of the DG and the SVZ bordering the lateral ventricles. Neurons formed in the SGZ appear to remain in the DG, and may mediate spatial memory^44^. In the present study, prenatal and early life TRAP exposure decreased adult neurogenesis in the DG, but not in the SVZ. Astrogenesis was not altered by TRAP in either region. This cell-type selectivity may arise during cell fate determination by Notch and Wnt signaling pathways, which promote differentiation of stem cells into astrocytes or neurons^45^. The regional selectivity of microglia activation in the DG is also notable because microglial activation can impair neurogenesis^46^. Environmental influences on neurogenesis are broadly considered to involve microglia^47^.

As mentioned above, microglia have critical roles in neurogenesis^46^. Activated microglia secrete factors that impair neurogenesis^48,49^. Specifically, TNF-α signaling through TNFR2 is required for neurogenesis, whereas activation of TNFR1 impairs neurogenesis^50^. We observed increased hippocampal TNF-α production following TRAP exposure by activation of the toll-like receptor 4 (TLR4) pathway in microglia^14^, and showed that impaired neurite outgrowth through increased levels of TNF-α in vitro, is dependent on TNFR1^51^. Moreover, microglial activation by TRAP produces extracellular reactive oxygen species and neurotoxic factors^52,53^. Chronic hippocampal microglial activation during development could contribute to deficits of behavior and neurogenesis from nPM exposure through direct neuronal interactions, as reported for gestational exposure to TRAP^32^.

Finally, we note an important difference of this TRAP-nPM exposure model from other developmental exposure models in its chemical fractionation of airborne PM. Two other exposure models cited above used concentrated ambient particles (CAPs, PM_0.1_)^31,33^ or diesel exhaust particles (DEP)^32,54^. In our protocol^20^, ambient PM_0.2_ are eluted from Teflon filters by sonication into distilled water. As noted above, the nPM yields particles for rodent exposure that are identical to ambient PM_0.2_ in their size distribution, content of WSOC, and inorganic ions, but are depleted in BC (BC > 10-fold) and water-insoluble organic carbon (WIOC)^20^. Thus, CAPs and DEP have higher proportions of BC and WIOC than TRAP-nPM, which may influence experimental outcomes: WIOC in PM0.2 includes PAHs, some of which are carcinogens, while others interact with steroid receptors^55^. The WSOC from Los Angeles includes organic acids, but only trace PAHs^56^. Nonetheless, the oxidative activity of WIOC and WSOC was approximately equivalent by the dithiothreitol (DTT) assay^57^. Because maternal PAH levels influence childrens’ myelination^4^, we anticipate that neurodevelopmental impact in rodent models will differ by the level of PAH exposure in these different experimental exposure paradigms.

## Conclusion

These data further demonstrate the importance of understanding the critical window of vulnerability during development and highlight the need for efforts to protect vulnerable populations. The adverse impact of pollution on the developing brain is likely to have life-long consequences that potentiate later neurodegenerative processes of aging. These findings support continuing government and community efforts to reduce urban air pollution. In particular, the location of public schools should be considered to minimize roadway pollution exposure.

## Electronic supplementary material

Supplementary legends

Supplemental Figure 1

Supplemental Figure 2

Supplemental Figure 3

## References

[1] Perera FP (2006). Effect of prenatal exposure to airborne polycyclic aromatic hydocarbons on neurodevelopment in the first 3 years of life among inner-city children. Environ. Health Perspect..

[2] Perera FP (2012). Prenatal polycyclic aromatic hydrocarbon (PAH) exposure and child behavior at age 6-7 years. Environ. Health Perspect..

[3] Guxens M (2018). Air pollution exposure during fetal life, brain morphology, and cognitive function in school age children. Biol. Psychiatry.

[4] Peterson BS (2015). Effects of prenatal exposure to air pollutants (polycyclic aromatic hydrocarbons) on the development of brain white matter, cognition, and behavior in later childhood. JAMA Psychiatry.

[5] Volk HE, Lurmann F, Penfold B, Hertz-Picciotto I, McConnell R (2013). Traffic-related air pollution, particulate matter, and autism. JAMA Psychiatry.

[6] Volk HE, Hertz-Picciotto I, Delwiche L, Lurmann F, McConnell R (2011). Residential proximity to freeways and autism in the CHARGE study. Environ. Health Perspect..

[7] Kalkbrenner AE (2018). Air toxics in relation to autism diagnosis, phenotype, and severity in a U.S. family-based study. Environ. Health Perspect..

[8] Allen JL (2014). Developmental exposure to concentrated ambient ultrafine particulate matter air pollution in mice results in persistent and sex-dependent behavioral neurotoxicity and glial activation. Toxicol. Sci..

[9] Davis DA (2013). Prenatal exposure to urban air nanoparticles in mice causes altered neuronal differentiation and depression-like responses. PLoS One.

[10] Bolton JL, Auten RL, Bilbo SD (2014). Prenatal air pollution exposure induces sexually dimorphic fetal programming of metabolic and neuroinflammatory outcomes in adult offspring. Brain Behav. Immun..

[11] Miller BR, Hen R (2015). The current state of the neurogenic theory of depression and anxiety. Curr. Opin. Neurobiol..

[12] Calderón-Garcidueñas L (2008). Long-term air pollution exposure is associated with neuroinflammation, an altered innate immune response, disruption of the blood-brain barrier, ultrafine particulate deposition, and accumulation of amyloid β-42 and α-synuclein in children and young adult. Toxicol. Pathol..

[13] Elder A (2006). Translocation of inhaled ultrafine manganese oxide particles to the central nervous system. Environ. Health Perspect..

[14] Woodward NC (2017). Traffic-related air pollution impact on mouse brain accelerates myelin and neuritic aging changes with specificity for CA1 neurons. Neurobiol. Aging.

[15] Fonken LK (2011). Air pollution impairs cognition, provokes depressive-like behaviors and alters hippocampal cytokine expression and morphology. Mol. Psychiatry.

[16] Padurariu M, Ciobica A, Mavroudis I, Fotiou D, Baloyannis S (2012). Hippocampal neuronal loss in the Ca1 and Ca3 areas of Alzheimer's disease patients. Psychiatr. Danub..

[17] Misra C, Kim S, Shen S, Sioutas C (2002). A high flow rate, very low pressure drop impactor for inertial separation of ultrafine from accumulation mode particles. J. Aerosol Sci..

[18] Moore K, Sheesley R, Schauer J, Sioutas C (2007). Daily variation in chemical characteristics of urban ultrafine aerosols and inference of their sources. Environ. Sci. Technol..

[19] Sardar SB, Fine PM, Mayo PR, Sioutas C (2005). Size-fractionated measurements of ambient ultrafine particle chemical composition in Los Angeles using the NanoMOUDI. Environ. Sci. Technol..

[20] Morgan TE (2011). Glutamatergic neurons in rodent models respond to nanoscale particulate urban air pollutants in vivo and in vitro. Environ. Health Perspect..

[21] Li N (2003). Ultrafine particulate pollutants induce oxidative stress and mitochondrial damage. Environ. Health Perspect..

[22] Verma V (2009). Redox activity of urban quasi-ultrafine particles from primary and secondary sources. Atmos. Environ..

[23] Sokolowski JD, Salamone JD (1994). Effects of dopamine depletions in the medial prefrontal cortex on DRL performance and motor activity in the rat. Brain Res..

[24] Simon NW (2013). Prefrontal cortical-striatal dopamine receptor mRNA expression predicts distinct forms of impulsivity. Eur. J. Neurosci..

[25] Balderas I (2008). The consolidation of object and context recognition memory involve different regions of the temporal lobe. Learn. Mem..

[26] Chehrehasa F, Meedeniya ACB, Dwyer P, Abrahamsen G, Mackay-Sim A (2009). EdU, a new thymidine analogue for labelling proliferating cells in the nervous system. J. Neurosci. Methods.

[27] Liu S (2014). Comparative analysis of H&E and Prussian blue staining in a mouse model of cerebral microbleeds. J. Histochem. Cytochem..

[28] Calderón-Garciduenãs L (2015). Air pollution and children: neural and tight junction antibodies and combustion metals, the role of barrier breakdown and brain immunity in neurodegeneration. J. Alzheimer’s Dis..

[29] Guxens M (2012). Prenatal exposure to residential air pollution and infant mental development: modulation by antioxidants and detoxification factors. Environ. Health Perspect..

[30] Volk HE, Lurmann F, Penfold B, Hertz-Picciotto I, McConnell R (2013). Traffic-related air pollution, particulate matter, and autism. JAMA Psychiatry.

[31] Klocke C (2017). Cory-Slechta consequences of gestational exposure to concentrated ambient fine and ultrafine particles in the mouse. Toxicol. Sci. J. Soc. Toxicol..

[32] Bolton JL (2017). Gestational exposure to air pollution alters cortical volume, microglial morphology, and microglia-neuron interactions in a sex-specific manner. Front Synaptic Neurosci..

[33] Church JS (2018). Perinatal exposure to concentrated ambient particulates results in autism-like behavioral deficits in adult mice. Neurotoxicology.

[34] Yokota S, Oshio S, Moriya N, Takeda K (2016). Social isolation-induced territorial aggression in male offspring is enhanced by exposure to diesel exhaust during pregnancy.. PLoS One..

[35] Oppenheim HA (2013). Exposure to vehicle emissions results in altered blood brain barrier permeability and expression of matrix metalloproteinases and tight junction proteins in mice. Part. Fibre Toxicol..

[36] Gregg NM (2015). Incidental cerebral microbleeds and cerebral blood flow in elderly individuals. JAMA Neurol..

[37] Akoudad S (2016). Association of cerebral microbleeds with cognitive decline and dementia. JAMA Neurol..

[38] Castagné, V., Moser P. & Porsolt R. D. Behavioral assessment of antidepressant activity in rodents. In: *Methods of Behavior Analysis in Neuroscience*. (Taylor and Francis, Boca Raton, 2009) pp 1–13. 21204330

[39] Allen JL (2013). Developmental exposure to concentrated ambient particles and preference for immediate reward in mice. Environ. Health Perspect..

[40] McEwen BS, Nasca C, Gray JD (2016). Stress effects on neuronal structure: hippocampus, amygdala, and prefrontal cortex. Neuropsychopharmacology.

[41] Toda T, Gage FH (2017). Review: adult neurogenesis contributes to hippocampal plasticity.. Cell Tissue Res..

[42] Freund J (2013). Emergence of individuality in genetically identical mice. Science (80-).

[43] Sorrells SF (2018). Human hippocampal neurogenesis drops sharply in children to undetectable levels in adults. Nature.

[44] Lazarov O, Hollands C (2016). Hippocampal neurogenesis: learning to remember. Prog. Neurobiol..

[45] Wen S, Li H, Liu J (2009). Dynamic signaling for neural stem cell fate determination. Cell Adh. Migr..

[46] Gemma C. & Bachstetter A. D. The role of microglia in adult hippocampal neurogenesis. *Front Cell. Neurosci*. http://journal.frontiersin.org/article/10.3389/fncel.2013.00229/abstract (2013). 10.3389/fncel.2013.00229 PMC3837350 24319411

[47] Valero J, Paris I, Sierra A (2016). Lifestyle shapes the dialogue between environment, microglia, and adult neurogenesis. ACS Chem. Neurosci..

[48] Ekdahl CT, Claasen JH, Bonde S, Kokaia Z, Lindvall O (2003). Inflammation is detrimental for neurogenesis in adult brain. Proc. Natl. Acad. Sci. USA.

[49] Monje ML, Toda H, Palmer TD (2003). Inflammatory blockade restores adult hippocampal neurogenesis. Science (80-).

[50] Chen Z, Palmer TD (2013). Differential roles of TNFR1 and TNFR2 signaling in adult hippocampal neurogenesis. Brain Behav. Immun..

[51] Cheng H (2016). Urban traffic-derived nanoparticulate matter reduces neurite outgrowth via TNFα in vitro.. J. Neuroinflamm..

[52] Block ML, Zecca L, Hong JS (2007). Microglia-mediated neurotoxicity: uncovering the molecular mechanisms. Nat. Rev. Neurosci..

[53] Block ML, Calderón-Garcidueñas L (2009). Air pollution: mechanisms of neuroinflammation and CNS disease. Trends Neurosci..

[54] Bolton JL (2012). Prenatal air pollution exposure induces neuroinflammation and predisposes offspring to weight gain in adulthood in a sex-specific manner. FASEB J..

[55] Chappell G, Pogribny IP, Guyton KZ, Rusyn I (2016). Epigenetic alterations induced by genotoxic occupational and environmental human chemical carcinogens: a systematic literature review. Mutat. Res. - Rev. Mutat. Res..

[56] Arhami M (2010). Organic compound characterization and source apportionment of indoor and outdoor quasi-ultrafine particulate matter in retirement homes of the Los Angeles Basin. Indoor Air.

[57] Saffari A, Daher N, Shafer MM, Schauer JJ, Sioutas C (2014). Seasonal and spatial variation in dithiothreitol (DTT) activity of quasi-ultrafine particles in the Los Angeles Basin and its association with chemical species. J. Environ. Sci. Heal - Part A Toxic./Hazard. Subst. Environ. Eng..

