# Supplementary material for: Prenatal and early life exposure to air pollution induced hippocampal vascular leakage and impaired neurogenesis in association with behavioral deficits
Source: Transl Psychiatry. 2018 Nov 29;8:261. doi: 10.1038/s41398-018-0317-1 (PMC6265287; doi:10.1038/s41398-018-0317-1)
Supplement: Supplementary file 1 — Supplementary legends [file 41398_2018_317_MOESM1_ESM.docx]

***Prenatal and Early Life Exposure to Air Pollution Induced Hippocampal Vascular Leakage and Impaired Neurogenesis in Association with Behavioral Deficits***

Abbreviated Title: Air pollution impairs hippocampal neurogenesis

NC Woodward^1^, A Haghani^1^, R Johnson^1^, T Hsu^2^, A Saffari^3^, C Sioutas^3^, SE Kanoski^2^,

CE Finch^1, 4^^, TE Morgan^1*^^

1, Leonard Davis School of Gerontology, University of Southern California, Los Angeles, CA.

2, Human and Evolutionary Biology Section, Department of Biological Sciences, University of Southern California, Los Angeles, CA; Neuroscience Program, University of Southern California, Los Angeles, CA.

3, Viterbi School of Engineering, University of Southern California, Los Angeles, CA.

4, Dornsife College, University of Southern California, Los Angeles, CA.

^contributed equally

*Corresponding Author: Todd E. Morgan, University of Southern California, 3715 McClintock Ave, Los Angeles, CA 90089-0191, Phone: 213-740-4083; email: temorgan@usc.edu

**Supplementary Figure 1**. nPM exposure did not alter body weight (A) or food intake (B). Fat mass was slightly lowerat 10 and 24 weeks (C). Lean mass (D) and fluid mass (E) were unaltered by nPM. *p<0.05

**Supplementary Figure 2:** nPM exposure decreased serum cytokines (A) IL-4, -25%, (B) IL-10, -25%, (C) IL-13, -20%. *p<0.05, **p<0.01

**Supplemental Figure 3:** No change was observed in anxiety behavior by elevated zero maze test, time spent in open arms. Right panel: Total arm entries were also unaltered by nPM exposure.
