# Supplementary figures and images for: Prenatal and early life exposure to air pollution induced hippocampal vascular leakage and impaired neurogenesis in association with behavioral deficits
Source: Transl Psychiatry. 2018 Nov 29;8:261. doi: 10.1038/s41398-018-0317-1 (PMC6265287; doi:10.1038/s41398-018-0317-1)

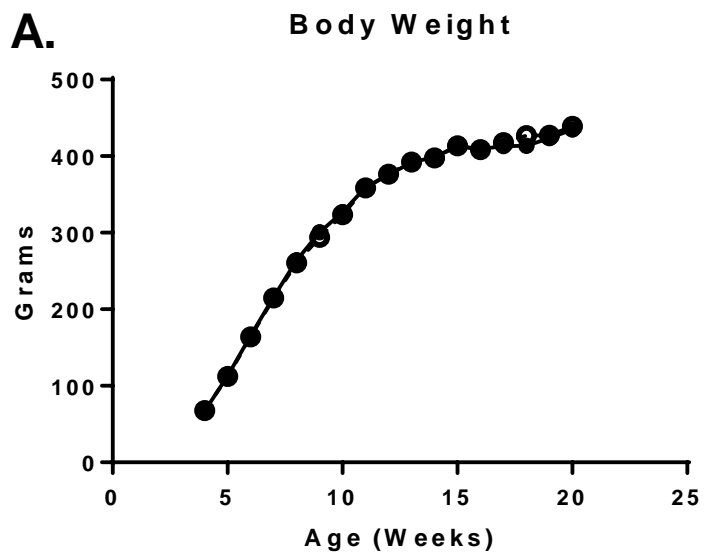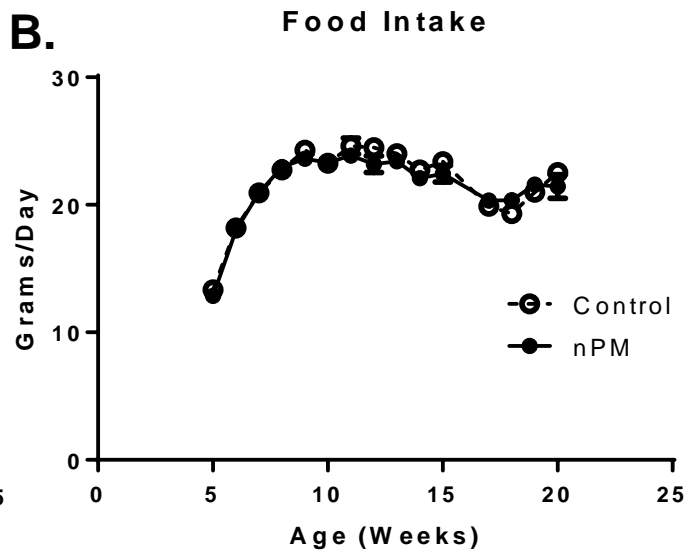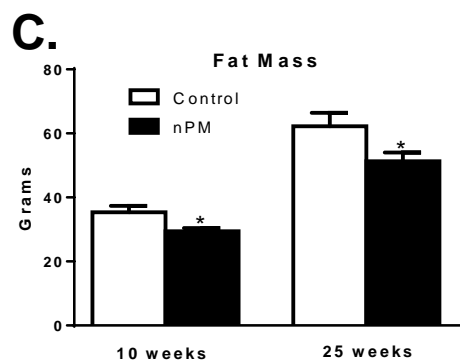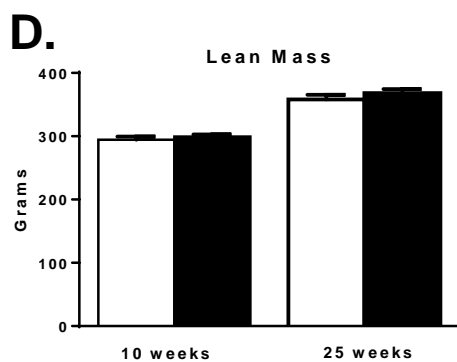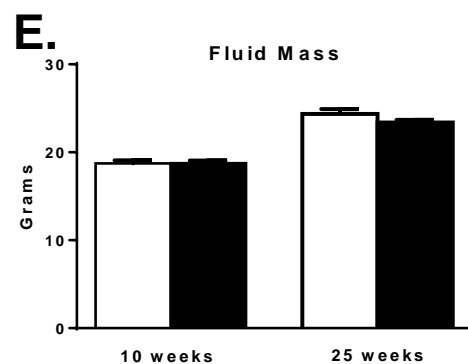

Supplement: Supplementary file 2 — Supplemental Figure 1 [file 41398_2018_317_MOESM2_ESM.pdf]

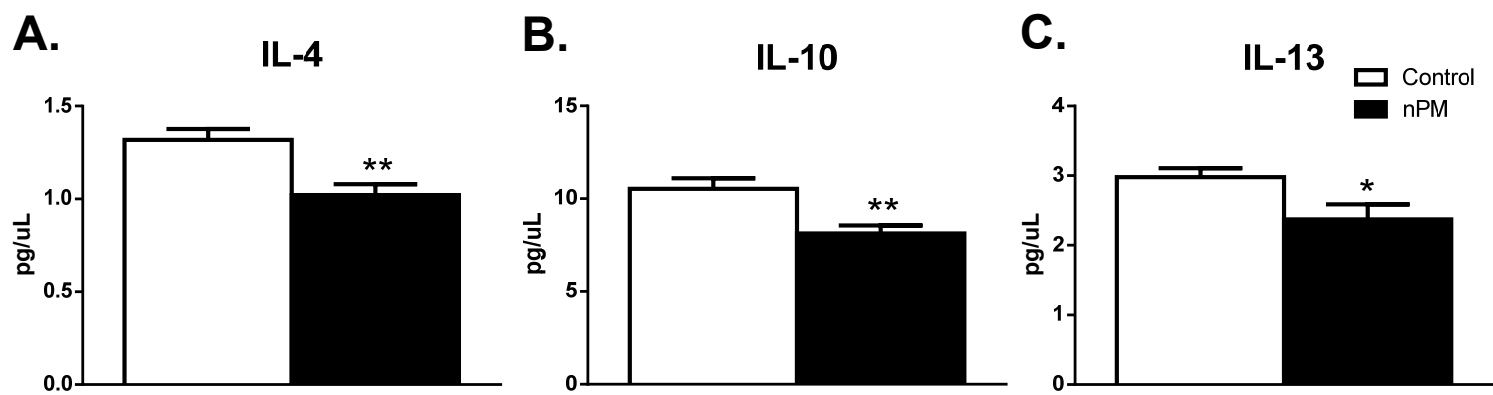

Supplement: Supplementary file 3 — Supplemental Figure 2 [file 41398_2018_317_MOESM3_ESM.pdf]

# Elevated Zero

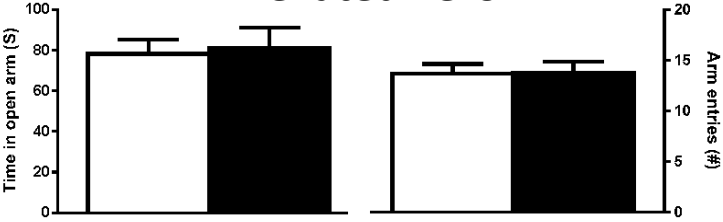

Supplement: Supplementary file 4 — Supplemental Figure 3 [file 41398_2018_317_MOESM4_ESM.pdf]
